# Biocompatibility of Chitosan Carriers with Application in Drug Delivery

**DOI:** 10.3390/jfb3030615

**Published:** 2012-09-17

**Authors:** Susana Rodrigues, Marita Dionísio, Carmen Remuñán López, Ana Grenha

**Affiliations:** 1 Centre for Molecular and Structural Biomedicine (CBME), Institute for Biotechnology and Bioengineering (IBB), Faculty of Sciences and Technology, University of Algarve, Campus de Gambelas, Faro 8005-139, Portugal; Email: susananasus@gmail.com (S.R.); maritadionisio@gmail.com (M.D.); 2 Department of Pharmacy and Pharmaceutical Technology, Faculty of Pharmacy, University of Santiago de Compostela, Campus Vida, Santiago de Compostela 15782, Spain; Email: mdelcarmen.remunan@usc.es

**Keywords:** biocompatibility, chitosan, cytotoxicity, genotoxicity, microparticles, nanoparticles

## Abstract

Chitosan is one of the most used polysaccharides in the design of drug delivery strategies for administration of either biomacromolecules or low molecular weight drugs. For these purposes, it is frequently used as matrix forming material in both nano and micron-sized particles. In addition to its interesting physicochemical and biopharmaceutical properties, which include high mucoadhesion and a great capacity to produce drug delivery systems, ensuring the biocompatibility of the drug delivery vehicles is a highly relevant issue. Nevertheless, this subject is not addressed as frequently as desired and even though the application of chitosan carriers has been widely explored, the demonstration of systems biocompatibility is still in its infancy. In this review, addressing the biocompatibility of chitosan carriers with application in drug delivery is discussed and the methods used *in vitro* and *in vivo*, exploring the effect of different variables, are described. We further provide a discussion on the *pros* and *cons* of used methodologies, as well as on the difficulties arising from the absence of standardization of procedures.

## 1. Introduction

Micron- and nanosized particles are sophisticated technologies that were developed to answer specific demands in the field of drug delivery, namely addressing the limitations posed by the administration of a new generation of low molecular weight drugs and biomacromolecules [1,2]. Chitosan and its derivatives in the last two decades have proven to be excellent and safe candidates for improving mucosal and trans-mucosal delivery or drugs, mainly due to their mucoadhesive and absorption enhancing properties, closely related with the cationic character of the polymer [3,4,5]. Indeed, due to its positive charge, chitosan has the special feature of adhering to mucosal surfaces, favoring the interaction of the drug with the mucus layer covering different epithelial surfaces [6,7,8]. The potential of chitosan for trans-mucosal drug delivery has been further strengthened by extensive demonstrations of its capacity, both *in vitro* and *in vivo*, in transitorily widening tight junctions between epithelial cells, thus facilitating the transport of poorly absorbable macromolecules through well-organized epithelia barriers [2,5]. This particular behavior governs the different toxicological patterns between chitosan and conventional absorption promoters, which are known to cause permanent epithelial damage. 

In addition to all these positive features, chitosan has been reported to exhibit other relevant properties, including biodegradability and biocompatibility [9,10,11]. In recognition of all these appealing characteristics, chitosan has been indicated as a promising biomaterial for biomedical and pharmaceutical, *i.e*., drug delivery, applications. The characterization of its biocompatibility pattern encompasses, therefore, a major issue, as it will drive the process of a future human drug delivery application. Whereas many of the technological applications of chitosan did not require regulatory approval, the use of chitosan in drug delivery, especially in particulate forms, requires significant improvement in documentation regarding the biocompatibility and safety of these products. Despite the outstanding reputation that comes with chitosan, it is true that a number of challenges related to biocompatibility have yet to be met; and some studies seem to raise some doubts concerning its applicability. Additionally, it is often erroneously assumed that the formulation of chitosan as a carrier does not have implications on its biocompatibility.

This review highlights the interest of biocompatibility assessment in the development of chitosan-based carriers for mucosal drug delivery. We provide the contextualization of biocompatibility in the field of drug delivery, presenting the methods used to assess this property and focusing on their advantages and disadvantages. Finally, the difficulties arising from the absence of standardized conditions of assessment are also addressed.

## 2. Chitosan Application in Drug Delivery

**Figure 1:**
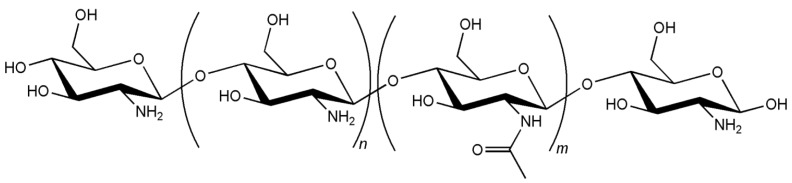
Chitosan structure (*n* and *m* assume different ratios) [14].

Chitosan is one of the most popular materials in the field of drug delivery and is, by far, the most applied of the natural polymers. Its attractiveness relies on very interesting structural and biological properties, which include the cationic character and the solubility in aqueous medium on one side, but most importantly, its characteristic biodegradability and mucoadhesivity on the other [4,6,10,12]. These properties are a result of the proper structure of the polysaccharide, which is composed of repeating alternated units of *N*-acetylglucosamine and D-glucosamine, linked by β-(1-4) glycosidic bonds [13], as depicted in Figure 1. 

Chitosan is available in a variety of forms that mainly differ in the molecular weight and degree of deacetylation [15,16]. These differences affect important characteristics such as solubility and mucoadhesivity [5]. With a p*K*_a_ of approximately 6.5, chitosan is soluble in acidic solutions owing to the protonation of the amino groups composing the polymeric chain at this pH [13,17]. In this regard, highly deacetylated chitosan (85%) is readily soluble in solutions of pH up to 6.5, but as the deacetylation degree decreases, the solubilization becomes more difficult [18]. With respect to the mucoadhesive capacity of the polymer, it is reported to increase with the increase in the deacetylation degree, as this provides more positively charged amino groups available for the interaction with negatively charged residues of the mucus, namely sialic acid [19,20]. In fact, this explains why the greatest part of chitosan applications in drug delivery reports the use of highly deacetylated chitosan, as mucoadhesion is responsible for a more prolonged retention in the site of action or absorption.

Applications of chitosan in the field of drug delivery have been mostly focusing on the production of carriers that improve the performance and effectiveness of encapsulated molecules, either macromolecules or low molecular weight drugs [12,21,22,23]. Nano and microparticles, as solid continuous matrixes, are the most usual carriers with a chitosan-based composition [4,23,24], but nanocapsules have also been reported [19,25]. Importantly, many works report the use of chitosan as a coating material, instead of being a part of the matrix of the system [22,26,27,28], which is a relevant recognition of chitosan appealing properties. In this respect, the coating of several different core structures has been reported, including solid lipid nanoparticles [26,29], polymeric nano and microparticles [28,30,31,32] and liposomes [33,34]. The clear objective of such an approach is to modify the surface properties of the core structure, either to improve the pattern of interaction with surrounding structures or to improve the biodegradation profile.

One of the most relevant properties of chitosan, with particular interest in drug delivery, relies on its ability to transiently open epithelial tight junctions. This capacity has been demonstrated on numerous occasions, both in the form of a solution and carrier, resulting in the permeation enhancement of macromolecules through well-organized epithelia such as the nasal [35,36], intestinal [37,38,39], ocular [40], buccal [41] and pulmonary [42]. Another characteristic of chitosan that increases interest in the polymer, is the flexibility of its molecular structure, which facilitates chemical modifications [43] that are performed to further improve several properties such as solubility or mucoadhesion [23,44,45].

Two of the most critical features of drug delivery systems concern their biodegradability and biocompatibility, which are mandatory requisites for acceptance by the regulatory agencies. Biodegradability becomes a crucial feature when considering acute and long-term toxicity, as non-degradable materials may accumulate in organs or even intracellularly [10]. Chitosan has been reported as highly biodegradable, which is mainly due to the fact that, under physiological conditions, its molecular chains can be digested by either lysozyme or chitinase. While the former is reported to exist in mucosal surfaces [10,11,46], the latter is produced by normal intestinal flora [11,43]. In addition, when oral delivery is under consideration, the hydrolytic activity of the acidic gastric medium has to be considered as an extra means of chitosan degradation [11,16]. 

However, the ability of a material to be biologically degraded, although positively understood, must also be considered from the point of view of the possibility of resulting in toxic degradation products [47]. In the case of chitosan, its degradation does not raise any critical concern, as the products of its metabolism are oligosaccharides that are either incorporated into glycosaminoglycan and glycoprotein metabolic pathways or easily excreted in urine directly [48,49,50]. Furthermore, evidence has been produced in several reports that no issues of accumulation/retention in the body are observed [10]. Nevertheless, if a too rapid degradation occurs, it might result in an accumulation of amino sugars, inducing an inflammatory response and, hence, affecting chitosan biocompatibility [51,52].

All the above mentioned properties of chitosan, from its intrinsic structural properties to the demonstrated mucoadhesive and permeation enhancement capacities, as well as biodegradability, endow this polymer with valuable potential for drug delivery applications. Altogether, these features have been resulting in a tremendously increasing number of publications and patents. The biocompatibility pattern of the polymer and its carriers is obviously of utmost importance to complete the background of relevant properties and to support the potential of chitosan, and will be addressed extensively in the following section.

## 3. Biocompatibility of Chitosan Carriers

**Figure 2:**
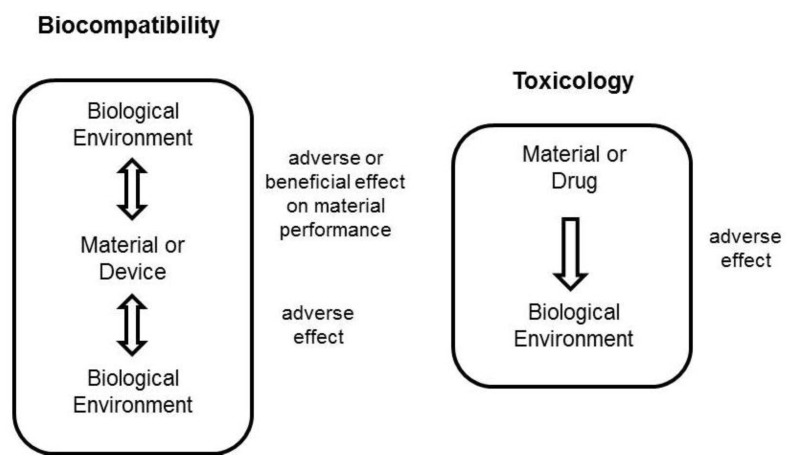
Illustration of the distinction between “biocompatibility” and “toxicity” [53].

As referred to previously, chitosan has been proposed very frequently as a carrier or functional excipient (for instance, as permeation enhancer) in the formulation of active compounds to be delivered [16]. Its biocompatibility is, thus, a current issue of great significance, although the real meaning of the word is many times disregarded. *Biocompatibility* is frequently addressed as *absence of toxicity*, but these are different concepts referring to different contexts. In a very elucidative review on the preclinical safety of polymeric carriers, *Gaspar and Duncan* clearly indicate that while drug molecules should be discussed in terms of toxicity, biomedical materials, which include polymeric materials, should be considered for their biocompatibility. From this perspective, and as depicted in Figure 2, toxicity refers to the potential harm that may be caused by a material, whereas biocompatibility further extends to the detrimental or beneficial effect of the physiological environment on the material performance [53]. 

This differentiation appears in the sequence of the first clear definition of biocompatibility agreed at a consensus conference of the European Society for Biomaterials [54] and the recent reformulation of that definition [55]. The first definition, dating from 1987, indicates biocompatibility to be the ability of a material to perform with an appropriate host response in a specific situation [54]. As said, this was recently re-defined to a more complete concept, as being the ability of a biomaterial to perform its desired function with respect to a medical therapy, without eliciting any undesirable local or systemic effects in the recipient or beneficiary of that therapy, but generating the most appropriate beneficial cellular or tissue response in that specific situation, and optimizing the clinically relevant performance of that therapy [55]. This comprehensive definition makes the contextualization of biocompatibility a mandatory requisite and addresses the possibility that materials are often required to specifically interact with the surrounding environment and in many cases with cell structures, instead of being ignored by them. It becomes, therefore, clear, that polymers (as well as polymer-based carriers) cannot be described as biocompatible without the proper contextualization of route of administration, frequency of administration and dose [53]. 

Actually, biocompatibility is both region- and host-dependent, meaning that different responses might be obtained when evaluating multiple sites or objectives [56]. In this respect, although when studies address these issues, the understanding of materials biocompatibility is hindered by the limited knowledge on the biological processes that are involved in material-cells interactions [56,57]. This is why several high-throughput technologies are being developed and applied to this end, examining global cell-biomaterial interactions in a faster way and addressing important questions such as the pathways and networks involved in cell-material interactions [56].

Almost all works concerning chitosan application refer to the polymer as a non-toxic, biologically compatible material, thus suitable to be used in carrier production in the field of drug delivery. However, although works addressing the biocompatibility of chitosan carriers are becoming more frequent, usually an incomplete set of assays is performed. In other cases, this question is completely disregarded, the authors simply assume polymer biocompatibility and, by affinity, that of the carrier. Sometimes this statement is based on chitosan approval by the American Food and Drug Administration (FDA) as a wound dressing material [16,21], for instance. It is a fact that chitosan is approved for that end and also as a dietary component in several other countries [16]. Nevertheless, and in the sequence of the above mentioned issues concerning the intrinsic definition of biocompatibility, chitosan biocompatibility must be addressed in the context of each particular case and condition. Accordingly, the FDA and other regulatory agencies do not approve materials in a general manner, instead evaluating and approving materials with respect to specific applications. In this context, a very important issue that seems to be overlooked on many occasions is that, as happens for many other materials, chitosan exists in several different structures and formulated as different types of carriers for administration in varied conditions. Each entity must, then, be considered separately, requiring specific testing in the particular conditions expected for its administration.

Still in this context, it is not infrequent to find works on the subject of carrier biocompatibility as being solely dependent on that of the material composing its structure. This is an erroneous approach, as several parameters, other than the material itself, are recognized mediators of the biocompatibility, affecting the carrier behavior and determining its clinical outcome; individual characteristics (age, sex, general health), as well as the proper structure of carriers (size, morphology, cristallinity, surface characteristics, degradation profile and products) are included in this list [55]. It is well recognized now that the physicochemical properties of materials can alter dramatically when formulated as specific structures like those of the different carriers, as particular interactions take place and the area available for contact with surrounding environments is modified in each case. Therefore, the carriers may exhibit new and unique biological properties, thus generating potential different risks as compared to the raw materials of the same chemistry [47]. 

The formulation of chitosan under the form of a carrier may have implications on its biocompatibility and, therefore, it is important to address, and treat differently, both the biocompatibility of chitosan as a molecule and as a carrier. As a positively charged molecule, chitosan usually provides a great interaction with cell membranes, which are negatively charged (approximately −70 mV) due to ionic interchanges between the intracellular and extracellular medium, which are mediated by the Na^+^/K^+^ pump [58]. Actually, owing to that positive charge, chitosan nanoparticles are often taken up by the cells [59]. When chitosan is formulated as a carrier and complexed with a drug, the number of positively charged amino groups that remains available is frequently decreased, comparatively. This lower number of charges consequently affects its capacity to interact either with cell membranes and the surrounding environment, potentially decreasing its uptake and, possibly, potential toxicity. As such, highly deacetylated chitosans have a naturally higher propensity for cell interaction, as has been demonstrated [60].

As previously mentioned, in parallel with the distinction between different chitosan types and the need to address the difference between molecules and carriers, it is of utmost importance that biocompatibility is understood as a contextual concept. This means that references to the biocompatibility of a carrier should be always accompanied by the intended application [55] or, at least, in the case of drug delivery, by the route of administration to be focused. 

As the carrier comes in contact with the host, a sequence of events is expected to take place, potentially including carrier–protein interactions, as well as those with other surrounding macromolecules, and also the induction of inflammatory and/or immune responses [55,56]. Therefore, assessing some of these events might give potential indications on the carrier biocompatibility. Many *in vitro* assays are frequently described to provide the evaluation of carrier biocompatibility, in many cases in accordance with ISO 10993 [61,62,63], which describes the topics of evaluation and testing relevant for the biological evaluation of medical devices. This standard indicates varied device categories, first dividing them according to the nature of their contact with the body (surface devices, external communicating devices and implants). Chitosan carriers as is the focus of this review are included in the category of surface devices. A second division then appears, addressing the contact time that is expected for the specific device with the body. The most basic tests considered for an initial evaluation refer to cytotoxicity, sensitization and irritation or intracutaneous reactivity. Tests should include the evaluation of cellular morphology and membrane integrity, as well as metabolism efficiency [61]. If contact time extends beyond 24 hours, acute or sub-acute toxicity must be also addressed (or even implantation tests, if applicable) [62]. Genotoxicity studies are also covered in the ISO 10993 [63]. The importance of assessing genotoxic effects relies on the fact that materials and carriers might not be cytotoxic, but evidence the ability to affect the genetic information of the cells they are in contact with, for instance leading to abnormal cell growth [64,65]. The standard also specifies that *in vitro* tests should be repeated several times, not only to ensure the validation of the used analytical methods, but also to address variations occurring in cells [61]. Furthermore, it designates, in certain conditions, a substitution of animal studies by specifically developed *in vitro* tests and indicates the need to improve animal tests to minimize animal pain and distress [66]. The tests performed using cells are generally devoid of ethical issues and are easier to control and reproduce, being also less expensive in comparison with studies involving animals [64]. Performing the proposed *in vitro* tests may not determine the final carrier biocompatibility, but will certainly comprise an important step towards animal studies and the final clinical trials. Importantly, performing this set of *in vitro* assays permits decreasing *in vivo* testing, thus complying with the requirements of the ISO.

In the following section, assays described in the literature for biocompatibility evaluation of chitosan carriers that are applied in drug delivery are presented.

### 3.1. *In Vitro* Cell Toxicity

*In vitro* tests of cell toxicity provide a rough assessment of the ability of cells relevant to a determined application to survive in the presence of specific materials or carriers, the latter being the subject of the present review. In general, two different protocols are observed, one providing a direct contact of the carrier with the cells, and another evaluating the effect of the contact with leachable materials (diffusible components, degradation products, *etc.*), thus considering an indirect cell contact [57,67]. 

A very wide number of assay techniques can be used to evaluate cytotoxicity. Table 1 describes those assays reported for the evaluation of chitosan carriers. Whatever the selected test, the generally desired outcome is that cell viability remains close to 100% after contacting with the tested material/system. Depending on which cellular characteristics are focused, the assays described to evaluate chitosan carriers are mainly divided into metabolic assays and membrane integrity assays. While the former assess occurrences of an early stage of cell death, the latter determine the occurrence of membrane disruption that is more frequent during the later stage of the process [68].

**Table 1:** Summary of *in vitro* cytotoxicity assays described for the evaluation of chitosan carriers.

Assay	Theoretical principle	Evaluated cellular function
Tryplan blue	Blue dye is excluded by viable cells	Cell membrane integrity
Propidium iodide	Red dye enters damaged cells and intercalates DNA, enhancing dye fluorescence	Cell membrane integrity
Lactate dehydrogenase	LDH leaks from damaged cell membrane. Enzyme transforms NADH + pyruvate into NAD^+^ + lactate:	Cell membrane integrity
	Direct quantification of NADH at 340 nm	
	Tetrazolium reduction to formazan	
Neutral red	Lysosomal uptake of red dye in live cells	Lysosomal membrane integrity
MTT, MTS, XTT	Tetrazolium reduction to blue formazan in metabolically active cells	Mitochondrial metabolism
Alamar blue	Resazurin reduction to pink resorufin by metabolically active cells	Mitochondrial metabolism

DNA: deoxyribonucleic acid; LDH: lactate dehydrogenase; MTT: 3–(4,5-dimethylthiazol-2-yl)-2,5-diphenyltetrazolium bromide); MTS: 3-(4,5-dimethylthiazol-2-yl)-5-(3-carboxymethoxyphenyl)-2-(4-sulfophenyl)-2H-tetrazolium); NAD: nicotinamide adenine dinucleotide; NADH: reduced form of nicotinamide adenine dinucleotide; XTT: (2,3-bis-(2-methoxy-4- nitro-5-sulfophenyl)-2H-tetrazolium-5-carboxanilide).

**Figure 3:**
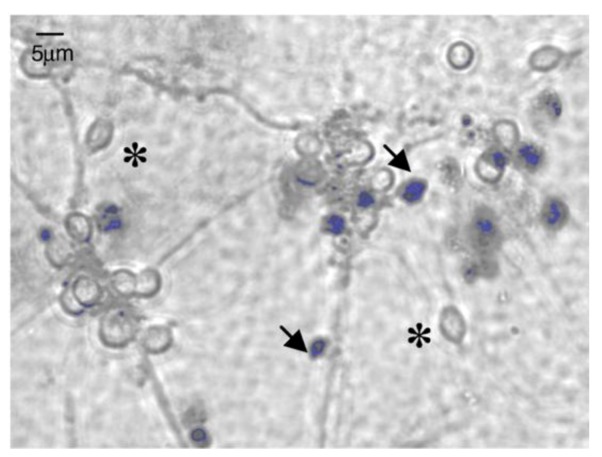
Illustration of the result of the Trypan blue exclusion assay on cell culture. Cells including trypan blue (dead cells) are stained in blue and marked with arrows. Adapted with permission from [74].

The cell membrane is a functional barrier, using several mechanisms to control the traffic of substances into and out of the cellular structure. A disrupted cell membrane implies cell death and, therefore, the assays providing the assessment of cell membrane integrity permit the discernment between live and dead cells. The trypan blue assay has been frequently used for this end, relying on the exclusion of the dye by viable cells, which possess intact cell membranes, while damaged cells include the dye because their membranes are no longer capable of controlling molecular permeation [64,69,70,71]. A viable cell thus exhibits a colorless cytoplasm, whereas a damaged one has a blue cytoplasm that is easily identified by microscopic observation, as is evidenced in Figure 3 [64,69,72]. Notwithstanding the easiness of the procedure, this assay has been referred as imprecise, leading to an overestimation of cell viability [69]. 

Propidium iodide, a red dye, is also used to indicate membrane disruption. It is membrane-impermeable owing to two positive charges existing in its structure [73], but it enters the cells when membrane damage occurs. It has a strong ability to intercalate DNA and registers a strongly enhanced fluorescence upon binding to nuclei acids, the fluorescence change being proportional to the number of damaged cells [64]. At an excitation of 488 nm, it gives a bright signal easily detected and quantified by flow cytometry. One of the problems with this assay is that the intercalation is reversible, so that the dye might leak out from cells that were dead before fixation and stain the previously viable cells [73].

Alternatively, an assay based on the determination of the amount of lactate dehydrogenase (LDH) that is leaking through a damaged cell membrane has also been used [70]. This enzyme is present in cell cytosol and catalyzes the reaction that transforms *NADH + pyruvate* into *NAD^+^ + lactate*. The increase of LDH activity in cell culture medium is proportional to the number of lysed cells. Two different protocols can be followed for the quantification of LDH. On one side, NADH can be directly quantified by spectrophotometry at 340 nm and its amount used to determine LDH level if the reaction starts with known levels of NADH and pyruvate [75]. On the other side, the most common approach uses commercial quantification kits based on a colorimetric assay. In these kits, the formation of NADH can be measured in a reaction where the tetrazolium salt INT is reduced to formazan, which is a colored substance. The amount of formazan is determined directly by spectrophotometry at 490 nm and proportionally correlates with the amount of LDH and, consequently, with the number of damaged cells [64]. The loss of intracellular LDH and its release into the culture medium is an indicator of irreversible cell death due to cell membrane damage [76,77]. 

The neutral red assay is one of the most frequently used, but it evaluates lysosomal membrane integrity rather than cell membrane integrity [71]. This assay is based on the principle that living cells are able to capture the dye, storing it in the lysosomes [64,67]. The efficiency of neutral red retention depends both on the pH of the lysosome and its membrane proton pump, which maintains the acid environment of the lysosomal compartment. Therefore, lysosomes in unstressed cells are able to retain the dye for longer periods and a destabilization of lysosomal membrane results in leaking of neutral red to the cytosol, decreasing the amount of dye retained in the cells. Neutral red is quantified by spectrophotometry at 540 nm [78]. 

Metabolic assays are also of widespread application, generally focusing the mitochondrial metabolism. A reduction of cellular metabolic activity is generally accepted as an early indicator of cellular damage. The most common assay, among all those evaluating cytotoxicity, belongs to this category and is the MTT test (uses the compound 3-(4,5-dimethylthiazol-2-yl)-2,5-diphenyltetrazolium bromide), which comprises the production of a dye by cells with active mitochondrial activity. This assay specifically evaluates an enzyme function, as the yellow tetrazolium salts are reduced to water-insoluble purple-blue formazan crystals by mitochondrial dehydrogenases. These crystals precipitate in the cytosol and are then solubilized after the induction of cellular lysis by a surfactant, enabling the absorbance to be read at 540 nm [67,76]. Variants of the assay include the MTS (3-(4,5-dimethylthiazol-2-yl)-5-(3-carboxymethoxyphenyl)-2-(4-sulfophenyl)-2H-tetrazolium) and XTT (2,3-bis-(2-methoxy-4-nitro-5-sulfophenyl)-2H-tetrazolium-5-carboxanilide) assay, which present the advantage of avoiding the solubilization step required in the MTT [64]. In all these assays, a dysfunction of mitochondrial activity is used as a sensor of disturbed cell function. A higher concentration of the dye relates to a higher amount of metabolically active cells, which is usually interpreted as higher cell viability. In some cases, this interpretation should not be as linear as it is taken generally, as the contact with a metabolic inhibitor would have the same effect without actually causing cell death. 

Alamar blue assay also falls in the category of MTT, using the same oxidation-reduction principle. The blue coloured agent contains resazurin that is reduced to pink resorufin by metabolically active mitochondria, being then quantified by fluorimetry with excitation at 545 nm and emission at 590 nm [71,79]. In both MTT (or variants) and Alamar blue assays, the reduction has been believed to be mediated solely by mitochondrial enzymes. However, some works have been suggesting that other enzymes present in the cytosol and microsomes can have a contribution to the reduction reaction [80,81]. 

Chitosan carriers have been frequently tested by means of the above mentioned assays, with a predominance of the MTT assay, not only using the original polymer [30,82,83,84], but also chitosan derivatives [85,86]. In some cases, a comparison is performed between chitosan solutions and chitosan carriers. In many works, however, the isolated effect of materials composing the matrix of drug delivery systems or that of drug-loaded carriers is not tested, the evaluation being performed only on empty carriers. In these cases, it is not possible to determine rigorously the systems behavior, because a material may not cause cell injury but kill an animal due to its drug release pattern [57]

When analyzing the available studies reporting the assessment of chitosan carriers’ biocompatibility, one easily observes that a huge variety of conditions are applied. Even if focusing specifically on the works related with drug delivery, a large number of different cells are used, varied material concentrations are applied, considering different contact times. In addition, the intrinsic variations provided by chitosan as a polymer cannot be disregarded (chitosan as a base or salt, different salts, molecular weight, deacetylation degree, *etc.*), not to mention that very different carriers are also available. To make the subject even more difficult, different assays are used that evaluate distinct aspects of cellular toxicity. This obviously translates into a wide range of responses that are practically impossible to compare, due to unstandardized conditions of assessment.

**Figure 4:**
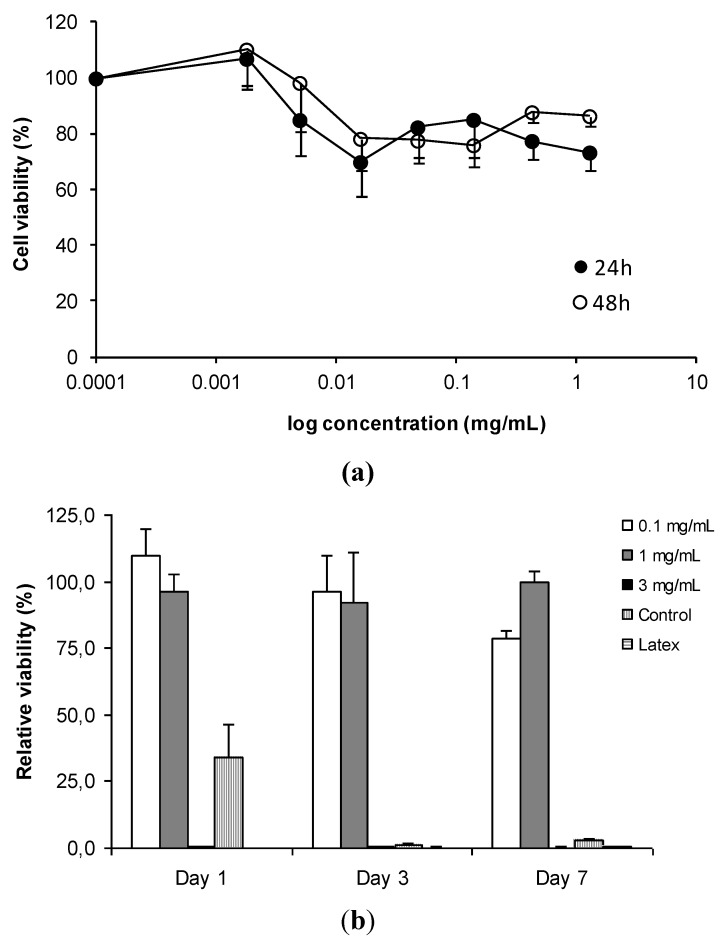
(**a**) Cell viability of chitosan/tripolyphosphate nanoparticles in Calu-3 cells (bronchial epithelial cells) determined by MTT assay (top graphic); and (**b**) chitosan/carrageenan nanoparticles in L929 cells (fibroblasts) determined by MTS assay (bottom graphic) [84,91].

Nevertheless, taking into account the overall information made available by the literature, it seems possible to say that the general trend indicates that in many cases chitosan solutions exhibit a certain degree of toxicity that is mostly dependent on the dose [60,87]. This confirms a fundamental principle of toxicology, first expressed by Paracelsus, saying that “The dose makes the poison”. Apart from depending on the dose, toxicity was also conditioned by the polymer characteristics, such as molecular weight and degree of deacetylation [60,88], as well as by the pH of the incubation medium and time of incubation [89,90]. In turn, it is also observed that when the polymer is used as matrix material of drug delivery systems, in most cases there is no overt toxicity in concentrations ranging up to approximately 1 mg/mL, although higher concentrations have been occasionally referred to not decrease cell viability as well [28,30,60,84,91,92,93]. However, the effect is still governed by the used dose [60,90,94]. Figure 4 provides examples of very low toxicity of different chitosan carriers determined using metabolic assays.

The different behaviors observed between chitosan molecules and carriers might be explained by the distinct conformation that molecular chains adopt when formulated as carriers, which does not allow the exposure of many of the groups responsible for interaction when in solution. Nevertheless, there are also works reporting a similar cytotoxic pattern for both chitosan solutions and particles [60].

This discrepancy of results is unfortunately observed in the literature and is attributed to the previously referred multitude of assay conditions that are used, not only focusing different structural materials and cells, but also in what concerns the general specificities of the assays. In the cases where chitosan is combined with other polymers to compose the systems’ matrix, the selected secondary polymer naturally affects the overall cytotoxicity of the carrier [95,96]. 

Many works also report the use of chitosan as a coating material [28,87,97,98]. These approaches can endeavor to achieve different outcomes, from an increased mucoadhesion, an enhancement of the biocompatibility profile by surface modification, or a general improvement in the formulation performance. Whatever the objective, the final biocompatibility of the system will obviously depend on its total composition. In this regard, it has been generally reported that no important toxicity is observed when chitosan is coating a specific core [87,97,99,100] or an improvement of the overall toxicity is obtained due to the presence of the polysaccharide [28,96,98]. 

**Figure 5:**
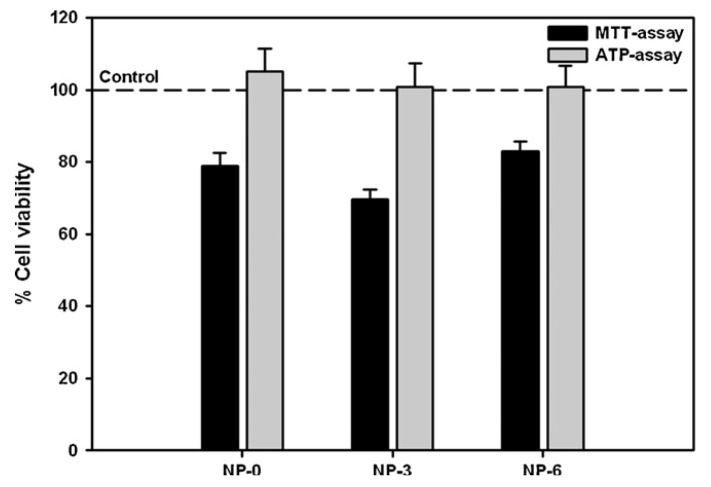
Comparison between the viability of A549 cells after incubation with different formulations of chitosan/PLGA nanoparticles (0.9 mg/mL) as determined by MTT and ATP assays [101].

One important question that is worth mentioning is that, whatever the selected assay, the samples incubated with the cells should be sterile, to avoid cell contamination and a misjudgment of cytotoxicity. It is further considered that conducting multiple tests, addressing distinct aspects of cellular function, is advantageous to ensure that valid conclusions are drawn, permitting a higher confidence on the observations [64]. An example of this strategy is evidenced in Figure 5, in this case with an observation of some differences in the quantification of cell viability, which are attributed to different sensitivities of the used tests.

### 3.2. *In Vitro* Detection of Impaired Cell or Epithelial Function

Although in some cases a toxic effect can result in cell death, there are many other subtler outcomes that do not cause sufficient harm to induce cell death, instead interfering in the normal functions or generating cellular irritancy or stress. In this context, several assays permit the monitorization of cell function. The assays performed in the ambit of the evaluation of chitosan carriers are described in Table 2. 

The adenosine triphosphate (ATP) assay assesses the functional integrity of cells, as cell injury results in a decrease of cytoplasmic ATP. ATP plays an important role in energy exchange in biological systems, serving as the principal immediate donor of energy and being present in all metabolically active cells, as any specialized function will demand the use of energy. ATP level can be determined by a luminescent assay in which an enzyme, luciferase, is used to catalyze the formation of light from ATP and luciferin. The emitted light intensity is determined and linearly related with the ATP concentration [101,102]. This assay is much more recent than others and, therefore, its use is not widespread. Works reporting its application indicate no alterations in ATP intracellular levels, both upon incubation with chitosan solution [103] and chitosan-based (polylactide-co-glycolide/chitosan) carriers [101]. This latter example is depicted in Figure 5.

**Table 2:** *In vitro* cell function and genotoxicity assays described for the evaluation of chitosan carriers.

Assay	Theoretical principle	Evaluated cellular function
ATP	Reduction in ATP cytoplasmic level indicates cell injury	Cell functional integrity
	Luciferase catalyses light formation from ATP and luciferin. Luminescence observation	
TER	Cellular damage or stress induces TER decrease	Cell barrier integrity
Comet	Electrophoresis separation of broken DNA strands which form the tail of the comet. DNA staining with dye and observation by fluorescence microscopy	DNA damage

ATP: adenosine triphosphate; DNA: deoxyribonucleic acid; TER: transepithelial electrical resistance.

**Figure 6:**
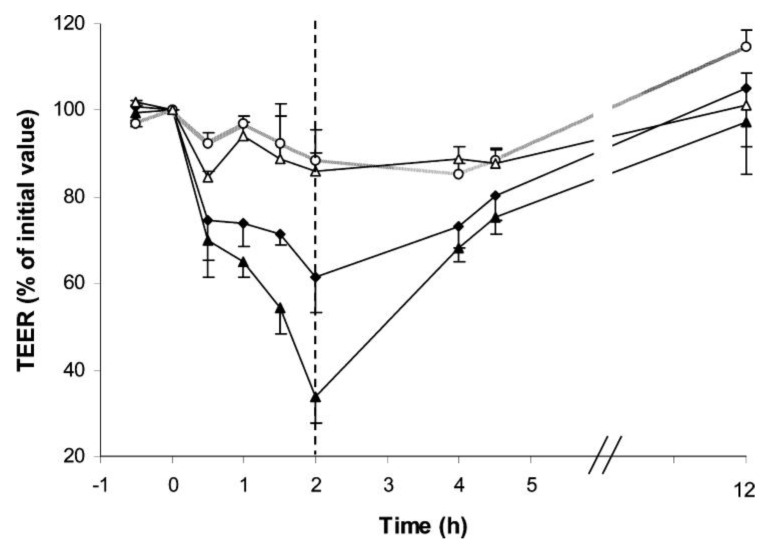
Effect of chitosan/cyclodextrin nanoparticles (40 μg/cm^2^) on the TER of Calu-3 cell monolayers at pH 6.4. Each point represents the mean ± SD (*n* = 5). Keys: (○) control HBSS pH 7.4; (Δ) control HBSS pH 6.4; (▲) chitosan/sulfobutylether-*β*-cyclodextrin/ tripolyphosphate (4/3/0.25) nanoparticles; (◆) chitosan/carboximethyl-*β*-cyclodextrin/ tripolyphosphate (4/4/0.25) nanoparticles; dotted line (----) represents the start of the reversibility experiment [115].

Chitosan carriers are often proposed in the ambit of systemic mucosal delivery of drugs, which means that the transported drug is expected to permeate an epithelial barrier and enter the blood circulation to be systemically distributed. Drug permeation through the transcellular pathway is dependent on several physicochemical properties of the drugs, namely on their lipophilicity. In turn, mucosal epithelia are generally characterized by the existence of a tight cell barrier, which evidences tight junctions between adjacent cells, so that the free diffusion of molecules by the paracellular route is prevented [104,105,106]. The intensity of the tightness of these junctions can be determined in cell cultures by a parameter called transepithelial electrical resistance (TER), which provides a measurement of the epithelial barrier properties and integrity [107,108,109]. TER has been pointed frequently as a sensitive marker for cellular damage, indicating sub-lethal toxicity [67,101,110] and any alteration in TER suggests a change in the epithelial barrier function [110]. Chitosan is known to have the ability to transiently open epithelial tight junctions, permitting an increase of drug permeation by acting as an absorption promoter. The mechanism underlying chitosan effect relies on the interaction of its protonated amino groups with cell membranes. This interaction produces a reversible structural reorganization of junction proteins, encompassing a specific redistribution of cytoskeletal F-actin and the tight junction protein ZO-1, which results in the junction opening [4,111]. With such an ability, chitosan and chitosan carriers have been frequently reported to improve the mucosal absorption of drugs through roughly all the routes of administration, contributing to increased bioavailability [21,22,87,112,113]. However, by the proper concept of permeation improvement by the mentioned mechanism, chitosan interferes with the normal barrier function of the epithelia. What makes this alteration acceptable is its non-permanent character, as the opening of tight junctions is known to be reversible when the contact with the polymer or the carriers is ceased [4,114,115,116]. An example of this effect is depicted in Figure 6, using chitosan/cyclodextrin nanoparticles. 

In the majority of works it is reported that the decrease of TER induced by chitosan is more pronounced for chitosan solutions than for chitosan carriers, which has been attributed to the fact that in the carriers there are less chemical groups exposed that are available for an interaction with cell surfaces, as compared with the polymer chains available in solution [117,118]. However, in some studies it has been observed that nanoparticles produced a more accentuated decrease of TER than solutions [116]. One possible explanation for these different observations is the fact that the carriers/solutions are in contact with the cells in media of different pH. As chitosan p*K*_a_ is around 6.5, the polymeric chains in solution or the carriers will form (or not) aggregates in dependence of the final pH of the medium they are incubated in. These aggregates will have different sizes, which promote different patterns of contact with the cells.

If considered that TER can be used to indicate alterations of cell function, as already explained, it is obvious that chitosan carriers evidence that effect. However, the demonstration that the effect is reversible, permitting a complete recovery of the cellular function upon removal of the stressing agent, is also a remarkable outcome. Although these observations cannot be used to say that chitosan carriers are biocompatible, the reversibility of the effect on the barrier integrity makes it being acceptable. 

### 3.3. *In Vitro* Genotoxicity

Genotoxicity assays provide an examination of the ability of chitosan carriers to damage cellular DNA upon contact with the cells. The most used test is called the comet assay (Table 2), which assays the DNA damage in individual cells using gel electrophoresis [64]. The cells are exposed to an electric field to attract the broken and negatively charged DNA to the anode. After that separation, a fluorescent dye is used to stain DNA, like propidium iodide. Afterwards, the gel is read and cells appear distributed as comets, with intact DNA residing in the head and broken DNA migrating to form the tail. The extent of the tail gives an indication on the number of DNA strand breaks [64,119].

Genotoxic evaluation of chitosan carriers is not frequent and a very scarce number of works are available. Chitosan oligosaccharides and a low molecular weight chitosan indicated an absence of genotoxicity in lymphocytes [120]. In a work with chitosan-coated silver nanoparticles proposed as an alternative to conventional antibiotics, the comet assay revealed that a concentration of nanoparticles of 3 ppm does not elicit a genotoxic effect on mouse macrophages (RAW264.7 cells). In contrast, 20 ppm of the nanoparticles induce the formation of a comet with a considerable tail [82].

### 3.4. *In Vitro* Monitorisation of Inflammatory Response

The development of an inflammatory response as a result of cell contact with carriers has also been used as an indicator of biocompatibility. Testing this hypothesis encompasses the quantification of the release of various inflammatory markers, namely the cytokines IL-6, IL-8 and the tumor necrosis factor (TNF)-α. While IL-8 is a chemotatic agent for inflammatory cells, IL-6 is responsible for neutrophil activation and TNF-α is an acute inflammatory response cytokine [121]. A limited number of works dealing with chitosan carriers for drug delivery applications refer to this evaluation. PLGA-CS nanoparticles demonstrated to not induce an increase in any of these inflammation markers upon incubation with Calu-3 cells in nanoparticle concentrations up to 0.2 mg/mL [96]. Chitosan microspheres encapsulating PLGA nanoparticles generally evidenced no ability to increase TNF-α secretion by RAW264.7 cells (macrophages) in concentrations up to 1.6 mg/mL [28]. The same cell line evidenced an absence of effect on the levels of IL-6, IL-8 and TNF-α mediated by the presence of chitosan-DNA nanoparticles up to 24 h, even at the higher nanoparticle concentration tested (60 µg/mL) [122].

As described for other tests, the highest limitation in performing a comparison of results lies in the variety of conditions, in this particular case most related in the wide range of concentrations tested. Interestingly, from concentrations as low as 0.06 mg/mL to much higher values (1.6 mg/mL) the observation remained very similar and reported no ability of the tested carriers to induce inflammatory responses.

### 3.5. *In Vivo* Studies

*In vivo* studies are essential for the final establishment of biocompatibility. Even when assuming that *in vitro* cell studies reflect the *in vivo* observations considering the same cells, it cannot be forgotten that in cell cultures only a few of the *in vivo* variables are accounted for, thereby not permitting a real simulation of the *in vivo* environment. In addition, cell cultures are very sensitive to environmental changes, such as temperature, pH and nutrient concentrations, demanding a confirmation of observations in an *in vivo* setting [64]. As critically pointed out in a recent review addressing the biocompatibility of drug delivery systems, a carrier may not cause any tissue injury at all, but kill an animal either from drug release or from problems related with intravascular coagulation, embolic events, chelation of ions vital to homeostasis, *etc.* [57]. This actually means that, although not directly cytotoxic, the carrier (as any material) can induce a destructive reaction thereafter [57], which makes *in vivo* assays mandatory and determinant after a certain moment and, definitely, well before any attempt of claiming for a human application of the developed carrier. This ambitious step needs to be accounted for regarding both the carrier effectiveness and safety.

Chitosan biodegradability represents a step forward concerning safety requirements, as it prevents its accumulation in the organism. As mentioned in Section 2, chitosan of suitable molecular weight is said to be directly cleared by the kidney, while that of excessive molecular weight is first degraded into fragments that undergo renal clearance afterwards [11,21]. As previously observed in some *in vitro* tests, chitosan carriers present a decreased toxicity profile as compared with the corresponding polymeric solutions, because the interaction pattern with cellular structures is different between free polymeric chains and the carriers. The same observation applies, in some cases, to the comparison between loaded and unloaded chitosan carriers. In fact, the loading of a carrier with a drug might cause surface alterations on the carrier, for instance at the level of its surface charge. Some works report that drug loading produces a charge decrease, thereby modifying the interactions with cells and the microenvironment, often leading to decreased toxicity [16]. However, the previous observation that drug release by itself can cause acute toxicity, depending on the release pattern, cannot be disregarded.

There are mainly two different *in vivo* studies that have been used to assess the biocompatibility of chitosan carriers. By one side it is important to observe histopathological effects resulting from the contact with the carrier. In turn, it is also relevant to determine the inflammatory response induced by the carrier, which is monitored by the determination of several pro-inflammatory cytokines. Additionally, the appearance of relevant alterations in normal clinical signs (diarrhea, fever or other systemic symptoms) is frequently monitored. In what concerns the evaluation of chitosan carriers, there is a high incidence in the assessment of both the oral and the pulmonary routes, but others have also been approached.

The LD_50_ of paclitaxel-loaded chitosan micelles administered intravenously to mice was 72.16 mg/kg. Moreover, intravenous administration to rabbits did not induce histophatological effects at a dose of 6 mg/kg/day during 3 days [123]. 

For the administration of carriers through the lung route, several different observations were performed using different particles. Chitosan-graft-spermine/pDNA nanoparticles administered to mice using a nose-only device revealed an absence of detectable damage. The histopathological evaluation of the lungs evidenced absence of necrosis, metaplasia, anaplasia in pneumocytes, atelectasis or emphysema. Capillary vessels within the alveolar wall were not enlarged and damaged endothelial cells were rarely observed. Neither congestion nor hemorrhage was noticeable, along with an absence of infiltration of inflammatory cells. In addition, abnormal features were not detected in other major organs (brain, heart, lung, liver, kidney and spleen) [124]. In contrast, the intratracheal administration of glycol chitosan/cholanic acid nanoparticles to mice (2 mg/kg) induced mild inflammation. Transient neutrophilic pulmonary inflammation was observed from 6 h to 3 days after administration and the lung expression of pro-inflammatory cytokines (IL-1β, IL-6, and TNF-α), as well as that of the chemokine MIP-1α, increased during the first 24 h, recovering to normal levels thereafter [125]. A more pronounced inflammatory effect was detected upon intratracheal administration to rats of unloaded chitosan microparticles (2–10 mg/kg of particles). A dose-dependent pro-inflammatory effect was manifested by increased levels of bronchoalveolar lavage fluid protein, lactate dehydrogenease activity and increases in lung tissue myeloperoxidase activity and leukocyte migration. A cytological examination of bronchoalveolar fluid further evidenced a large infiltration of neutrophils, which are inflammatory cells [126]. Unfortunately, the authors did not include any group of animals examined sometime after the inhalations, to verify whether a recovery from the inflammation occurred.

It is also noticeable that in the latter work the used dose varied from 2 to 10 mg/kg, while the previous one only assessed a dose of 2 mg/kg. Obviously the different composition of the particles in both works has a great impact on the observed response, apart from the fact that the former work assesses nanoparticles, while the latter tests microparticles. However, it is important to mention that chitosan microparticles administered at the dose of 2 mg/kg induced a response similar to that of the control in practically all the assayed parameters [126], thus being considered to not induce an inflammatory response. When comparing both systems assessed at the same concentration (2 mg/kg), a similar absence of inflammatory effect was determined.

For the oral administration varied responses were also reported. An evaluation of the hepatotoxicity of 3 doses (125 mg/kg each dose) of unloaded alginate/chitosan microparticles administered to guinea pigs spaced by 10 days, revealed no toxic effect by measurement of the indicators of liver function serum bilirubin, alanine aminotransferase and alkaline phosphatase [127]. Unloaded chitosan/poly-γ-glutamic acid nanoparticles were reported to not induce significant toxicity upon oral administration of a daily dose of 100 mg/kg for 14 days. As compared to untreated animals, there was no evidence of different clinical signs. In addition, no pathological changes in liver, kidney and intestinal segments were observed [128]. The ability of these nanoparticles to induce hepatotoxicity was also verified by monitoring alanine aminotransferase and aspartate aminotransferase. No alteration in the parameters was obtained after the administration, nor was a histological effect observed [129]. Trimethyl chitosan oligomer/DNA nanoparticles administered orally twice a week during 4 weeks, caused slight diarrhea at 3 weeks in animals treated with 0.5 mg/mL of nanoparticles (containing 0.1 mg/mL DNA), which was relieved upon stopping the administration [130]. This was considered very mild toxicity. 

A very small increase of serum cytokine levels (IL-2, IL-6, IL-12, TNF-α) was observed upon administration of chitosan and polyethylenelglycol-coated PLGA nanoparticles (20 mg/mL) to mice, which completely recovered to normality at 6 h [131]. Chitosan reduced gold nanoparticles demonstrated a very high LD_50_ (greater than 2000 mg/kg) after administration to rats for a 28 day period. No significant changes were detected in clinical signs, body weight, food consumption, hematological parameters, organ weights or histopathological aspects [132].

Although occasionally some augmented inflammatory response has been reported, the general trend seems to be that no important toxicity or only minimal toxic effects are generated by the administration of chitosan-based carriers. This might be a significant indicator of chitosan safety, but ascertaining the specific conditions to be established in each administration modality and, somehow, provide a standardization of the procedures, remains the most important issue. 

## 4. Conclusions and Expert Opinion

Biocompatibility is not a simple interaction between one material and one cell type, but may instead involve degradation products, separate effects from drug and carrier, as well as numerous cell types that present themselves at different times. Therefore, mitigating problems with biocompatibility is rather challenging as, apart from the material properties, poorly understood drug- and material-tissue interactions are also involved. Carriers are also becoming increasingly complex, composed of multiple materials, demanding the need to find components that may yield any toxic influence on the carrier as a whole. In turn, as immune reactions are so complex and individual, the biocompatibility of a carrier cannot be adequately described only by taking into account a single type of cell or tissue. 

The most common assays testing biocompatibility of chitosan carriers are *in vitro* cytotoxicity assays, which are usually performed for short periods, rarely going beyond 96 h and in most cases remaining in the 12–24 h or even less. As a result, these assays only measure finite effects on cells during the first hours after exposure to toxic substances. After contact with a toxic or stressing agent, the cells either recover from, or succumb to, the induced injury. Therefore, in addition to the typical protocol of cytotoxicity evaluation that includes cell incubation with the carrier and an immediate measurement of the parameter being tested, it would be useful to remove the carrier and permit cell incubation with culture medium for a certain period, for instance 24 or 48 h, before performing the cytotoxic evaluation. The ISO 10993 [61,62,63] gives a good indication of the type of tests that should be considered initially, indicating that an early test of carrier toxicity might avoid expensive and undesirable development work. However, the assertive evaluation of toxicity will only be possible when standard conditions are implemented, so that all reported results converge from well-defined assay settings. In this way, comparing the panoply of results would be a much more accurate and facilitated process and higher benefit would be taken from the produced data. 

Another important aspect is that most of the conducted studies are performed in immortalized cell lines but, at least after obtaining satisfactory toxicity profiles, it would be important to repeat those studies in primary cell cultures, as these closely resemble the *in vivo* environment and are, therefore, more realistic [133]. Different sensitivity has been observed between primary and established cell lines [37,38] and, although primary cell cultures are more difficult to establish, they register variability among different donors, which will contribute to the similarity of *in vivo* situations, where inter-individual variability is also observed. In summary, the *in vitro* studies of biocompatibility should assess both the carrier and the drug and, when necessary, the individual components of the carrier. Furthermore, different cells lines, as well as primary cell cultures, should be used with adequate time periods and likewise incubation/recovery protocols, tested. 

Notwithstanding the importance of *in vitro* testing at the initial stage of evaluation of a determined carrier, it is known that the resulting findings frequently lack predictive value of *in vivo* occurrences. *In vivo* assays therefore become essential. As mentioned for the *in vitro* studies in cell culture, a wide variety of assay conditions is also observed in *in vivo* tests. This disparity focuses not only the performed tests, but also the protocols of each test. Some authors assess the loaded carriers, while others test unloaded particles. Different media are used to suspend the particles, when applicable. Some authors monitor the animals for a certain period after the last administration, in order to assess the reestablishment of normal functions; while others do not assess this possibility. Therefore, once again a standardization of procedures would be helpful and essential to allow advancements in the evaluation of chitosan carriers.

As previously mentioned, chitosan is available in a wide range of different characteristics (molecular weight, deacetylation degree, several different chemical derivatives). It must be, then, recalled that different polymeric characteristics might result in structurally different carriers and, consequently, in diverse responses *in vitro* and *in vivo*. Therefore, different carriers and raw materials should be tested accordingly. Considering the available literature on the subject as a whole, chitosan carriers have generally demonstrated to not exhibit overt toxicity, but actually neither nanoparticles nor microparticles can be seen as inert. Microparticles are generally taken up by macrophagic cells very easily, thus having the potential to initiate an inflammatory reaction. In turn, the biological consequences of nanoparticle interactions are not well understood so far, but they should be addressed cautiously, considering their ability to gain a size-dependent access to intracellular compartments [57]. Nevertheless, it is interesting to observe, for instance, that chitosan microparticles (around 150 µm) received approval as a support for the growing of fibroblasts [134], an observation that reinforces evidence of chitosan potential.
